# Road Dust Lead (Pb) in Two Neighborhoods of Urban Atlanta, (GA, USA)

**DOI:** 10.3390/ijerph9062020

**Published:** 2012-05-25

**Authors:** Daniel M. Deocampo, Jack Reed, Alexander P. Kalenuik

**Affiliations:** 1 Department of Geosciences, Georgia State University, Atlanta, GA 30303, USA; Email: preed2@gsu.edu; 2 A.P. Nicholas Environmental Contractors, LLC, 151 Locust St., Avondale Estates, GA 30002, USA; Email: paul.kalenuik@apnicholas.com

**Keywords:** Pb, geochemistry, geospatial, road dust, children’s health

## Abstract

Road dust continues to be a major potential reservoir of Pb in the urban environment, and an important potential component of child Pb exposure. This study presents ICP-AES analyses of metals in 72 samples of road dust (<250 µm) collected in the urban core of Atlanta, Georgia. In the Downtown area, median Pb concentrations are ~63 mg/kg Pb, with high values of 278 mg/kg. For comparison, median Pb values in a nearby residential neighborhood (also in the urban core) were ~93 mg/kg, with a high of 972 mg/kg. Geospatial variability is high, with significant variation observed over tens to hundreds of meters. Spearman Rank Correlation tests suggest that Pb and other metals (Cu, Ni, V, Zn) are associated with iron and manganese oxide phases in the residential area, as reported in other cities. However, Pb in the Downtown area is not correlated with the others, suggesting a difference in source or transport history. Given these complexities and the expected differences between road dust and soil Pb, future efforts to assess exposure risk should therefore be based on spatially distributed sampling at very high spatial resolution.

## 1. Introduction

Significant progress has been made worldwide over the past half century in reducing child Pb poisoning rates. In the US, for example, estimates of children and pregnant women with high blood Pb levels have dropped significantly in recent decades [1]. Despite this progress, about 250,000 children in the US have blood lead levels over the current Level of Concern of 10 µg/dL [2]. This number is likely to double as the U.S. Centers for Disease Control and Prevention (CDC) considers the recommendation of its advisory body to lower the Level of Concern from 10 µg/dL to 5 µg/dL [3]. This major change in national policy is based on a large and growing body of evidence showing that even single-digit blood Pb levels have significant impacts on Intelligence Quotients, Attention Deficit Hyperactivity Disorder risks, cardiovascular disease, and kidney function [4,5,6,7,8]. If CDC finalizes this change, the World Health Organization will likely consider a similar move, potentially impacting the Pb regulatory environment worldwide and improving the prospect of reducing the Pb burden on millions of impacted children.

The most common Pb exposure pathways for children are ingestion or inhalation of Pb-bearing particulate matter, whether in the household or outdoor environment [9,10,11]. The most common sources of Pb are paint (typically as lead chromate, PbCrO_4_ or lead carbonate, PbCO_3_) [12,13] and gasoline containing tetraethyl lead. In many settings, mining and smelting operations remain major sources as well [14]. Chronic exposure is a long-term legacy, even in jurisdictions where lead releases were minimized or eliminated long ago, because Pb persists in near-surface soil environments [15,16]. In the US, socio-economic analyses have shown that the exposure burden is disproportionately carried by lower-income and minority communities, although significant risks to upper income and non-minority populations are found as well [1].

Numerous studies have shown the importance of the soil Pb reservoir in the cycling of Pb in the urban environment [16,17,18,19,20,21]. Depending on climate, soil Pb can be seasonally resuspended, leading to predictable quantitative exposure rates among children [15,16]. In highly urbanized environments, however, assessing soil Pb content can be difficult because of high spatial variability and confounding factors such as landscaping, erosion, and the presence of impervious surfaces. In a number of cities, studies of road dust have also been undertaken, as this material is present in significant quantities and is also susceptible to resuspension [21,22,23,24]. 

The purpose of this paper is to report the preliminary results of analyses of road dust metal contents in two urban neighborhoods of Atlanta, GA, USA [25]. The working hypotheses of the study are that: (1) despite strict controls on emissions, Pb persists in Atlanta road dust; and (2) physical and social processes in different neighborhoods produce different geospatial patterns in metal load.

## 2. Materials and Methods

In May, 2011, two neighborhoods were sampled near the heart of the urban core of Atlanta. The first area is known locally as “Downtown”, and is comprised largely of high-rise buildings in a commercial setting. The second area is comprised of the western two thirds of Atlanta’s Neighborhood Planning Unit V (NPU-V). The neighborhood is within 1 km of Downtown, and is a residential neighborhood with mostly early to mid-20th century construction; the neighborhood has a history of mixed residential and light commercial use. Both neighborhoods are adjacent to major transportation arteries. Seventy two (72) samples of road dust were collected by sweeping a 1 m × 1 m square area and collecting the dust in plastic bags. The two neighborhoods were selected because they are in the urban core of Atlanta, but they have different land uses. Individual sample sites were located randomly in a grid across the area, with added complexity of problems of access and several samples that did not contain enough fine material for analysis. These samples were excluded from the analysis. All analyzed samples are reported here.

In the lab, samples were sieved at 250 µm, and shipped to ALS Chemex (Reno, NV, USA) for geochemical analysis. Samples were pulverized and digested in nitric, perchloric, hydrofluoric, and hydrochloric acid, and analyzed by inductively coupled plasma atomic emission spectrometry. Analytical precision is in all cases less than 10% of the reported values. Metal abundances were treated as non-parametric data; correlations among the analytes were determined by Spearman Rank Correlation coefficent calculated using SPSS (v. 18). Geospatial analysis of the results was done using the Geostatistical Analysis tool in ArcGIS Desktop.

## 3. Results

### 3.1. Concentrations

Road dust Pb concentrations are highly variable in both neighborhoods (Table 1). In the Downtown area, Pb ranged from 25 to 278 mg/kg, with a median value of 63 mg/kg (n = 26). In NPU-V, Pb ranged from 17 to 972 mg/kg, with a median value of 93 mg/kg (n = 48). The median value for the combined dataset is 85 mg/kg (n = 74); the commercial downtown area therefore has somewhat lower road dust Pb concentrations compared to the residential neighborhood. Mapping of road dust Pb concentrations Downtown shows more or less uniform concentrations throughout the area, with the exception of two high values found in the northwest (Figure 1A). In NPU-V, road dust to the south tends to have higher Pb concentrations (as high as 452 mg/kg) compared to the north where Pb concentrations are near the median (Figure 1B).

**Table 1:** Summary of metal analyses of road dust in Atlanta, Georgia. All units in mg/kg.

	# analyses	Minimum	25th percentile	Median	75th percentile	Maximum
**Downtown**	**26**					
Cu		20	53	70	137	226
Cr		80	119	161	193	310
Ni		6	11	16	20	68
Zn		63	128	204	344	789
Pb		25	52	63	98	278
**NPU-V**	**48**					
Cu		22	49	68	113	1,445
Cr		76	109	129	171	385
Ni		7	14	18	22	74
Zn		68	143	220	308	1,115
Pb		17	65	93	142	972

**Figure 1:**
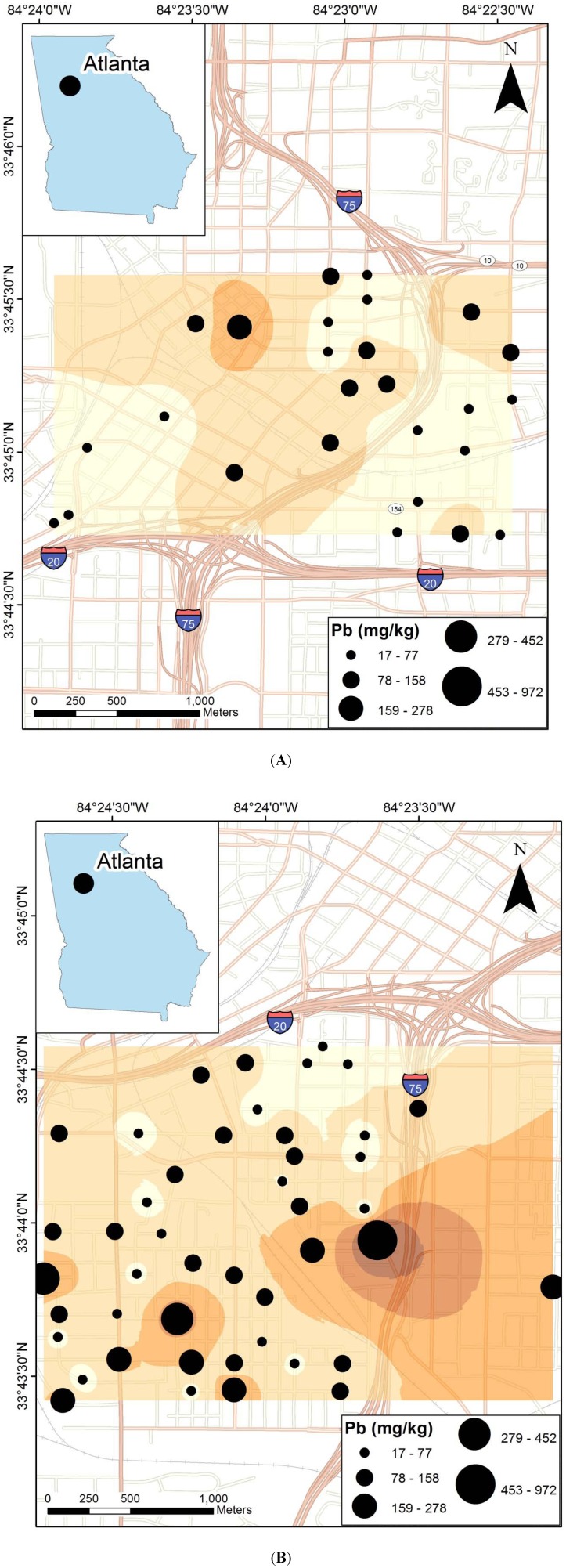
Maps of road dust Pb concentrations and interpolated prediction values in (**A**) Downtown Atlanta, and (**B**) Neighborhood Planning Unit V (NPU-V).

### 3.2. Intercorrelation

**Figure 2:**
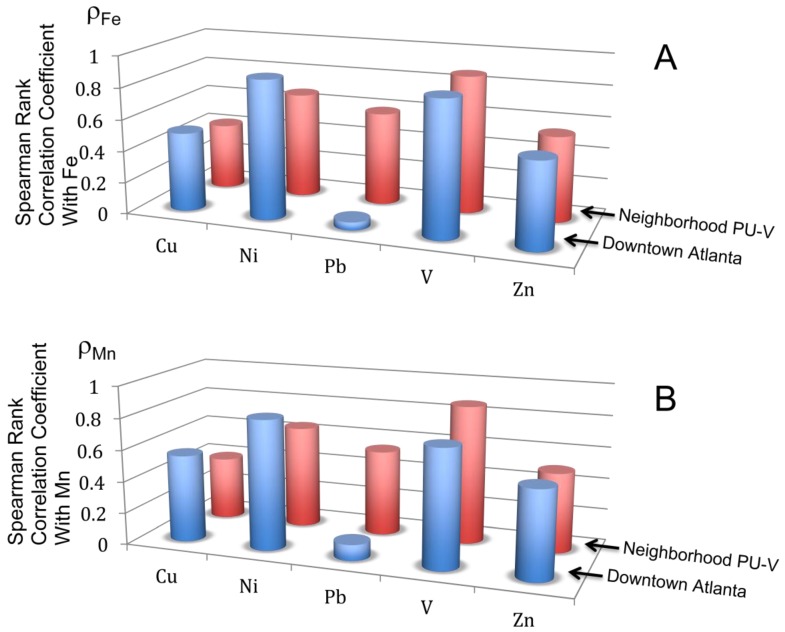
Spearman Rank Correlation Coefficients of metals with Fe (**A**) and Mn (**B**). Downtown, no relationship is found between Pb and the other metals, whereas in the residential NPU-V, the relationships are strong.

Several significant (*p* < 0.005) Spearman Rank correlations are found among several metals (Table 2 and Table 3). Downtown, Pb is not correlated with any other metal; however, it is well correlated with Co, Cu, Fe, Mn, Mo, Ni, and P in NPU-V. This major difference in Pb correlation is shown in Figure 2. In the Downtown area, all of the metals shown (Cu, Ni, Pb, V, and Zn) have significant Spearman Rank Correlation coefficients with Fe and Mn. However, in NPU-V, Pb does not correlate, even though the others do.

**Table 2:** Matrix of Spearman Rank Correlations of all available analyzed elements in road dust in Downtown Atlanta, GA, USA.

	Ba	Co	Cr	Cu	Fe	Mn	Mo	Ni	P	Pb	S	V	Zn
**Ba**		**–**0.01	**–**0.15	0.48	0.13	0.16	0.12	0.21	**–**0.04	0.01	0.34	**–**0.10	0.29
**Co**			0.27	0.36	**0.70**	**0.59**	0.28	**0.73**	0.45	0.10	**0.59**	**0.55**	0.40
**Cr**				0.30	0.36	0.25	**0.65**	0.44	0.18	0.07	0.14	0.24	0.37
**Cu**					**0.50**	**0.55**	**0.71**	**0.72**	0.32	0.23	**0.56**	0.24	**0.65**
**Fe**						**0.88**	0.42	**0.88**	**0.69**	0.05	**0.69**	**0.85**	**0.54**
**Mn**							0.35	**0.82**	**0.71**	0.10	**0.71**	**0.74**	**0.55**
**Mo**								**0.60**	0.39	0.21	**0.44**	0.25	**0.73**
**Ni**									**0.63**	0.12	**0.74**	**0.69**	**0.61**
**P**										0.19	**0.75**	**0.75**	**0.72**
**Pb**											**–**0.12	**–**0.05	0.42
**S**												**0.62**	**0.63**
**V**													0.36
**Zn**													

## 4. Discussion

### 4.1. Statistical Analysis

The lack of strong correlation with other metals in the Downtown road dust is surprising, and contrary to results found in other studies, especially those that focused on soil Pb [26]. The association of Pb from automobile exhaust with other metal indicators of automobiles provides some statistical evidence regarding the source. In other studies where soil Pb concentrations are heavily impacted by transportation infrastructure (*i.e.*, fuel exhaust legacy), associated deposits are often rich in metals such as Zn and Cu that have other automobile sources such as tire or brake dust [27]. 

In a chemical speciation study, Wang *et al*. [28] showed that metals in road dust in London and Hong Kong were highly associated with Fe-Mn oxides, with typically >70% of the Pb associated with Fe-Mn oxide phases. Li *et al*. [22] found similar results, as did Lu *et al*. [29]. The Atlanta results are consistent with this finding for the residential neighborhood, where Spearman Rank Correlation coefficients with both Fe and Mn are 0.5 or greater for Cu, Ni, Pb, V, and Zn (Figure 2). In Downtown Atlanta, the other metals are also highly correlated with Fe and Mn, with the exception of Pb. This suggests a reservoir of Pb in Downtown road dust other than Fe-Mn oxides; perhaps carbonate species originating from industrial paint sources, which are not regulated as residential paints are. Another possible source that would deliver exclusively Pb without much other metal contribution is wheel weights, which are ~95% Pb [30].

### 4.2. Geospatial Distribution

Initial prediction maps were constructed using various approaches including ordinary kriging and inverse distance weighting. However, autocorrelation analysis using Moran’s I (ArcGIS for Desktop 10 Advanced) revealed no discrete spatial correlation; therefore a weighted geospatial prediction map was not appropriate [31]. The prediction maps shown in Figure 1 are not weighted, but are based on a simple inverse distance weighting interpolation, and should only be considered an approximation of the Pb distribution pattern. 

Road dust samples in the northwest of the Downtown area have elevated Pb concentrations (Figure 1A). The Downtown area has median road dust Pb of ~63 mg/kg. The high values in the northwest therefore represent enrichment of three to four times the median Downtown values, signifying a potential source in that direction.

**Table 3:** Matrix of Spearman Rank Correlations of all available analyzed elements in road dust in residential Neighborhood Planning Unit V, Atlanta, GA, USA.

	Ba	Co	Cr	Cu	Fe	Mn	Mo	Ni	P	Pb	S	V	Zn
**Ba**		0.23	**–**0.13	0.18	0.15	0.22	0.16	0.33	0.24	0.14	**0.51**	0.12	0.35
**Co**			0.19	0.29	**0.75**	**0.84**	0.16	**0.63**	**0.61**	**0.45**	0.27	**0.81**	**0.50**
**Cr**				0.34	0.42	0.30	**0.49**	**0.47**	0.05	0.39	0.21	0.24	0.36
**Cu**					0.42	0.40	**0.57**	0.60	0.24	**0.55**	0.30	0.29	**0.62**
**Fe**						**0.85**	0.16	**0.67**	**0.62**	**0.59**	0.24	**0.87**	**0.54**
**Mn**							0.18	**0.65**	**0.71**	**0.54**	0.44	**0.87**	**0.50**
**Mo**								**0.56**	0.07	**0.45**	0.39	**–**0.06	**0.58**
**Ni**									0.51	**0.68**	0.44	**0.52**	**0.82**
**P**										**0.54**	**0.59**	**0.70**	**0.59**
**Pb**											0.35	0.39	**0.79**
**S**												0.30	**0.55**
**V**													0.35
**Zn**													

In NPU-V, high Pb content is found in the road dust in the southern portion of the sampled area, reaching a maximum of 972 mg/kg. A steep north-south gradient is observed (Figure 1B). In addition to the overall greater concentrations of Pb found in the south of NPU-V, the range of values is greater in the south. Road dust Pb in the NPU-V is not in all cases higher than that found Downtown, but discrete enriched areas are found, leading to overall a higher median Pb value for NPU-V. Intervening areas have road dust Pb more comparable to that of Downtown. Although the typical association between Pb and Zn is found in NPU-V (Table 3), which is commonly interpreted as representing a transportation-related source of the Pb, no obvious traffic corridor parallels the east-west trend of the high in the south portion of NPU-V (Figure 1B). 

The complexities in the road dust Pb distribution reflect the complex natural and anthropogenic processes at play in the road environment. In addition to the potential complexities of Pb source location across space and time (*i.e.*, transportation patterns and land use), road dust Pb is more susceptible to remobilization compared to soil Pb. Mobilization and transport of road dust Pb is not well understood, but the relatively impervious surface of roadways would make transport easier by both wind and episodic water action (e.g., stormwater sheetwash). It seems likely that impervious surfaces and a lower levels of organic and mineral matter make road dust Pb more mobile than soil Pb. This may account for the significant drop found in studies of Hong Kong road dust between 1986 and 2000 [22]. Over time, then, the effects of banning Pb in gasoline may be reflected by progressively lower Pb content in road dust; soil Pb, however, is not as likely to lower over time because Pb retention is higher in soils [16].

## 5. Conclusions

Road dust in the NPU-V and Downtown Atlanta neighborhoods has median Pb concentrations of about 85 mg/kg. Values in the northwest of the Downtown area reach a high of 278 mg/kg Pb, whereas high samples in the southern part of NPU-V reach 972 mg/kg Pb. These results suggest that despite the complexities of resuspension and transport of Pb-bearing particulates by wind and water in the road environment, substantial Pb remains in road dust decades after Pb-containing gasoline was banned, just as it is well known to persist in urban soils. This may be due to limits on rates of Pb removal from road dust, or other continuing sources of Pb in the urban environment. Geospatial variation can be observed at the scale of tens to hundreds of meters, and probably at even finer scales. Very high resolution of sampling is therefore required to adequately assess Pb exposure risks due to road dust, as well as other sources of Pb in the urban setting.
